# Association between Variation in Body Fat Mass Magnitude and Intake of Nutrients, including Carbohydrates, Fat, and B Vitamins, in a Cohort of Highly Trained Female Athletes

**DOI:** 10.3390/foods12224152

**Published:** 2023-11-16

**Authors:** Marius Baranauskas, Ingrida Kupčiūnaitė, Jurgita Lieponienė, Rimantas Stukas

**Affiliations:** 1 Faculty of Biomedical Sciences, State Higher Education Institution Panevėžys College, 35200 Panevėžys, Lithuania; ingrida.kupciunaite@panko.lt (I.K.); jurgita.lieponiene@panko.lt (J.L.); 2 Department of Public Health, Institute of Health Sciences, Faculty of Medicine, Vilnius University, 01513 Vilnius, Lithuania; rimantas.stukas@mf.vu.lt

**Keywords:** body fat mass, B vitamins, carbohydrates, fat, female athletes, food composition, nutrition

## Abstract

The most common sports nutrition strategies were constructed not only for maximizing musculoskeletal adaptations to exercise, but also to minimize health risks in athletes. Given the lack of research highlighting the potential effects of the intake of carbohydrates, fats, and B vitamins on body fat percentage in a population of female athletes, this study aimed to elucidate whether the intake of macronutrients and B vitamins could be associated with the variation in body fat percentage in a cohort of professional female athletes. This cross-sectional study was weighted to represent Lithuanian elite female athletes (n = 89). The dietary assessment of the female athletes was carried out using a 3-day dietary recall method. Their body composition was assessed using the bioelectrical impedance analysis method. For females, the reported average intakes of energy, carbohydrates, protein, and fat were 2475 kcal/day, 5.1 g/kg/day, 1.5 g/kg/day, and 36.7%, respectively. Excess B vitamin intake was revealed, ranging within plus 1–2 standard deviations (SDs) around the mean requirements. As a consequence, excessive body fat percentage was potentially factored as a negative outcome in maintaining optimal body composition in female athletes. Multivariate logistic regression analysis of a sample of female athletes revealed that, despite a slightly positive energy balance (∆ 95 kcal/day), the carbohydrate-deficient diet (adjusted odds ratio (aOR) 0.3, 95% confidence interval (CI) 0.1; 0.7), along with higher intakes of vitamin B_1_ (aOR 2.9, 95% CI 2.6; 7.8), vitamin B_2_ (aOR 6.7, 95% CI 1.1; 8.3), and vitamin B_3_ (aOR 1.8, 95% CI 1.4; 7.8) from food, was associated with a lower percentage of body fat. Therefore, more attention should be given to the intake of B vitamins in professional athletes with a range of body fat mass percentages for the purpose of achieving long-term goals of maintaining body composition and fitness.

## 1. Introduction

From the point of view of public health, people need to respond adequately to their nutritional needs in order to reduce the health-related risks of overweight and obesity [1]; thus, many nutritional recommendations have been proposed by healthcare professionals for the entire population at the global level [2,3]. Also, specific nutrition guidelines for the athletic population have been established due to a significant increase in physical loads and an increase in bodily energy demands [4]. The most common sports nutrition strategies were constructed not only for maximizing musculoskeletal adaptations to exercise, but also to minimize health risks in athletes [5].

In the present context, it should be noted that increased levels of physical activity have a meaningful impact on nutritional needs, particularly in terms of the intake of macronutrients and energy. Some sports make it difficult for athletes to consume the right quantity and type of food at the appropriate time. Thus, the nutrition goals for athletes should rely on eating foods of adequate quality and quantity, with the intention of full replenishment of energy expenditure, to avoid sport-related malnutrition leading to exercise-induced fatigue [4]. Additionally, individual requirements for the intake of both macronutrients and micronutrients could vary depending on numerous factors, including not only the type of sport or age, but also possible sex disparities associated with the health status, physiology, and body composition of athletes [6,7,8,9,10,11]. More specifically, female athletes have a higher risk of low energy availability [9] and the athletic triad [8,9], and differ from male athletes in terms of their metabolic profiles [11], hormonal specificity (i.e., higher estrogen) [8,12], and body composition characteristics [13,14,15]. Female athletes are prone to greater body fat contents compared to male athletes [13,14,15].

Taking into consideration the fact that body composition, as a significant vector, can impact health status [16,17,18,19] and the efficiency of sports performance [20,21], a more supportive body composition profile (e.g., relatively lower fat percentage) could be beneficial for female athletes, especially for those playing weight-sensitive sports [13,22].

Nutritional strategies related to diets that are lower in calories and/or carbohydrates for optimizing the muscle-to-fat ratio are usually used in sports routines [23,24,25,26,27,28,29,30,31,32,33,34,35]. Some studies highlighting the effects of low-carbohydrate–high-fat diets on body composition have proposed that even with a large weight reduction, lean body mass is maintained and body fat mass is significantly reduced [36,37]. However, Valsdottir et al. reported controversial results indicating that the low-carbohydrate–high-fat diet had no superior effect on female body composition compared to a normal diet, with or without exercise [38]. Additionally, regardless of the impacts of macronutrients such as carbohydrates and fat on body fat mass levels, little is known about the relationship between specific B vitamins and body composition extremes in athletes. Vitamin B’s key role in energy and lipid metabolism, triggering inflammatory responses and oxidative stress, and developing a higher risk for obesity, has been documented by scientific research [39,40,41,42,43]. However, while some observational studies have found a relationship between B vitamins—namely, B_1_, B_2_, B_6_, B_9_, and B_12_—and obesity [44,45,46], other studies showed no connection between B vitamins and body fat levels in non-athletic populations [47].

Given the inconsistency of the reported results in the entire population and the lack of research highlighting the potential effects of the consumption of carbohydrates, fat, and B vitamins on the body fat percentage in a population of female athletes, our study focused on exploring these associations and providing new evidence-based scientific results.

The aim of this study was to elucidate whether macronutrients and B vitamin intake could be associated with variations in body fat percentage in a cohort of professional female athletes.

The main objectives of this research were as follows:

(1) To assess body composition in a sample of elite female athletes.
(2) To evaluate the overall nutritional status of professional female athletes.
(3) To investigate a possible relationship between nutritional intake and the magnitude of body fat mass in high-performance female athletes.

Thus, this study examined the following research questions (RQs) (Nomenclature table):

**RQ1.**  *Do low-carbohydrate, high-fat diets relate to a lower body fat percentage?* 

**RQ2.**  *Are vitamin B (B_1_, B_2_, B_3_, B_6_, B_9,_ and B_12_) intakes above the recommended dietary allowances (RDAs) associated with lower body fat in highly trained female athletes?* 

## 2. Materials and Methods

### 2.1. Study Participants

**Figure 1:**
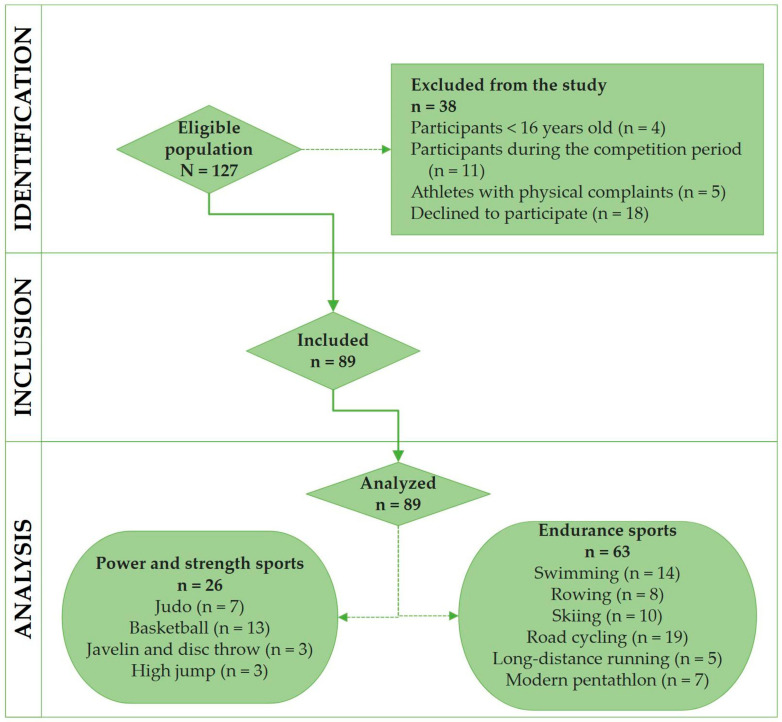
Study flowchart with exclusion criteria.

A total of 127 female athletes of an eligible population were selected from a target population comprising 361 Lithuanian elite athletes. The representative sample size of 96 cases, by considering a 95% two-sided confidence interval (CI), was calculated [48]. The probability sampling method was applied for the selection of the sample from the athletic population. Figure 1 provides more accurate information on the study enrollment process.

The participant inclusion criteria were as follows: (a) female athletes; (b) athletes who competed on a regular and organized basis; (c) participants of national and international competitions (candidates for the Olympics); (d) participants in the preparation phase; (e) participants who regularly followed a 6-day workout schedule plan for a week (training > 12 h a week); (f) participants who trained under four-year preparation programs derived from The National Olympic Committee; (g) participants taking no medications. The exclusion criteria were set as follows: (a) participants < 16 years old (junior athletes); (b) participants in the competition period; (c) athletes with physical complaints; (d) females without menstrual periods; (e) athletes who declined to participate in the study. 

### 2.2. Body Composition Assessment

The measurements of height (m) and the core components of body composition were performed following a 4 h fasting window with no participation in moderate-intensity physical activities and with no alcohol and/or caffeine intake within 24 h [49]. All of the assessments were carried out by the same sports nutritionist at the Lithuanian Sports Medicine Center. Body composition was analyzed using the bioelectrical impedance approach referring to the third level of validity [50,51]. The multi-frequency bioelectrical impedance analysis method with frequencies ranging from 1 kHz to 1000 kHz was applied to predict both the intracellular and extracellular fluid values of the study participants. Thus, body mass (BM, kg, and %), body fat mass (BF, kg, and %), fat-free mass (FFM, kg, and %), and skeletal muscle mass (SMM, kg, and %) were estimated via an X-scan device (International Organization for Standardization adopted by the European Union (EN-ISO): 13488, Seoul, South Korea, https://www.iso.org/home.html (accessed on 15 November 2023)). The readings of the BIA device were generated from the specs of the impedance device, where, for commercial purposes, the equations were concealed from potential users.

The estimated percentages of both FFM (%) and SMM (%) were compared to the normative propositions set for females [52]. The FFM normative values for females range from 70% to 80%, while the SMM percentages vary between 64 and 80%. The BF percentages for female athletes fluctuate within ranges from <15% to 30–34%: ‘too low’ (<15%), ‘lean’ (15–19%), ‘optimal’ (20–24%), ‘acceptable’ (25–29%), and ‘excessive’ (30–34%). In addition, the muscle-to-fat ratio (MFR) of female athletes was evaluated by dividing SSM (kg) by BF (kg). The female athletes’ MFRs were graded based on ranges that specified an ‘insufficient’ MFR (<1.89), a ‘too small’ MFR (from 1.9 to 2.89), a ‘moderate’ MFR (from 3 to 3.99), an ‘extensive’ MFR (from 4 to 5), and a ‘maximum’ MFR (>5) [52].

### 2.3. Dietary Intake Assessment

A dietary assessment of female athletes was conducted using a standardized 3-day dietary recall method [53,54,55,56]. Detailed information regarding the total food amounts and beverages consumed by female athletes was obtained through structured interviews conducted by the sports dietician from the Lithuanian Sports Medicine Center. Participants were asked to provide details about the amounts of food they consumed and consider the size of portions by using plates, bowls, slices, tablespoons, cups, teaspoons, and grams in accordance with the Atlas of Food [57]. Female athletes were encouraged to provide recipes for the meal dishes they had consumed. Finally, energy and dietary macro- and micronutrient intakes were evaluated via the Nutrition Baseline Software (NutriSurvey, SEAMEO-TROPMED RCCN-University of Indonesia, http://www.nutrisurvey.de/ (accessed on 5 April 2018)) [58]. The standard food database of NutriSurvey was manually complemented with the nutrition values of food items retrieved from the Lithuanian chemical composition tables [59].

The estimated energy requirement (EER) was calculated, aiming to identify the energy needs of female athletes [60]. Therefore, the Harris–Benedict equation [61] was applied to predict the resting metabolic rate (RMR). Furthermore, the training energy expenditure (TEE) was calculated in line with the guidance of the American Dietetic Association [4] and the Compendium of Physical Activities [62] developed to standardize the assignment of metabolic equivalent (MET) intensities in physical activity.

Nutritional adequacy was rated by comparing nutrient intakes to reference values (dietary reference intakes (DRIs)) established for planning and assessing diets for healthy athletes [63,64,65]. More specifically, for female athletes, the recommended dietary allowances (RDAs) for carbohydrate, protein, and fat intakes corresponded to 7–10 g/kg/day, 1.4–2.0 g/kg/day and 25–35%, respectively [63,64]. Given that an increased intake of micronutrients has little or no ergogenic effect on the athletic population, the consumption of vitamins and minerals should meet the RDAs documented for a target country. Consequently, an average daily B vitamin (B_1_, B_2_, B_3_, B_6_, B_9_, B_12_) intake among female athletes should be sufficient to meet only the RDAs [65]. On the condition that the RDAs were set at the estimated average requirements (EARs) plus twice the standard deviations (SDs) (RDA = EAR + 2 SD) [66], both deficiencies and excesses in the dietary intake of nutrients in the sample of female athletes were identified in the cases where the mean dietary macro- and micronutrient intakes (after comparing them to the RDAs) fluctuated above minus or plus 2 SDs around mean requirements, respectively.

### 2.4. Statistical Analysis

The body composition outcomes, namely, BM, BF, FFM, SMM, and MFR, along with the intakes of energy, macronutrients (carbohydrates, protein, and fat), and micronutrients (vitamins B: B_1_, B_2_, B_3_, B_6_, B_9,_ and B_12_) were presented as the means ± SDs. Welch’s *t*-test (for unequal variances) was applied to compare the mean intensities of the body composition outcomes between the female athletes, who were allocated to subgroups of power and strength sports as well as endurance sports. Nutritional adequacy was tested by comparing nutrient intakes to the RDAs. A one-sample *t*-test was used to determine whether there was a significant difference between the means of the two groups (intakes of energy and essential nutrients vs. RDAs). Furthermore, Cohen’s D (*d*) estimates as effect sizes were calculated to support the results obtained from the *t*-tests. The *d* values were interpreted as follows: 0 ≤ |*d*| < 0.2 (‘trivial effect’), 0.2 ≤ |*d*| < 0.5 (‘small effect’), 0.5 ≤ |*d*| < 0.8 (‘moderate effect’), 0.8 ≤ |*d*| (‘large effect’), 1 ≤ |*d*| < 2 (the difference between the two means was larger than 1 SD), 2 ≤ *d* (the difference between the two means was larger than 2 SDs) [67].

Two multiple linear regression models, A and B, were obtained to assess the relationship between MFR as a dependent variable and independent variables such as BF (in kg) and SMM (in kg) in the cohorts of female athletes competing in power and strength sports or endurance sports. 

Finally, a multivariate logistic regression model was constructed to calculate the adjusted odds ratios (aORs) and 95% confidence intervals (CIs) and to reveal whether an association exists between the intakes of essential nutrients and body fat mass as a dependent variable in female athletes. The dependent variable, namely, BF (in kg and %), of female athletes (23% ≥ |BF| > 23%) was converted to the dichotomous form (0—BF was below the median; 1—BF was equal to or larger than the median (reference category)). The units of the measurement of independent variables, namely, the intakes of energy (kcal/day), macronutrients (g/kg/day, %/day), and vitamin B (mg/day, mcg/day), were recorded to the dichotomous form, depending on the cut-off values that were calculated using the medians. The logistic regression model was adjusted to the sports branches of the study participants. The goodness-of-fit of the logistic regression model was evaluated using the Nagelkerke R^2^ (R^2^_N_) statistic. All the analyses were performed via Statistical Package for the Social Sciences (IBM SPSS Statistics) version 25 (Armonk, NY, USA), and a two-sided *p*-value < 0.05 was considered statistically significant. Statistical visualization was performed using Microsoft Visio Professional 2021 (Microsoft Corporation, Redmond, WA, USA).

## 3. Results

### 3.1. Sample Characteristics

This cross-sectional study pooled 89 professional female athletes aged 18 to 31 (20.9 ± 2.8 years) who were engaged in endurance sports (n = 63) as well as in power and strength sports (n = 26), including swimming, rowing, skiing, road cycling, long-distance running, modern pentathlon, judo, basketball, javelin and disc throw, and high jump. All the female athletes were recruited only during the preparatory phase. The sports exercises were performed 5.9 ± 0.7 days per week, while the mean duration of workout sessions was 172.1 ± 55.2 min a day.

As shown in Table 1, BM amounted to 62.0 ± 10.5 kg for female athletes. The means ± SDs of FFM, SSM, BF, and MFR for the female athletes were 76.9 ± 4.2%, 43.8 ± 5.5%, 23.0 ± 4.2%, and 3.2 ± 0.8, respectively. 

The results of the *t*-test disclosed significant differences (*p* < 0.05) associated with higher levels (in %) of BM, FFM, SMM, and BF in the subgroup of females from power and strength sports compared to those engaged in endurance sports. 

The MFR (2.9 ± 0.8) of females from strength and power sports was significantly lower than the MFR (3.4 ± 0.8) of females from endurance sports, as was predicted by higher BF levels only classified as ‘acceptable’ (25–29%) for optimal athletic performance (*p* < 0.05).

**Figure 2:**
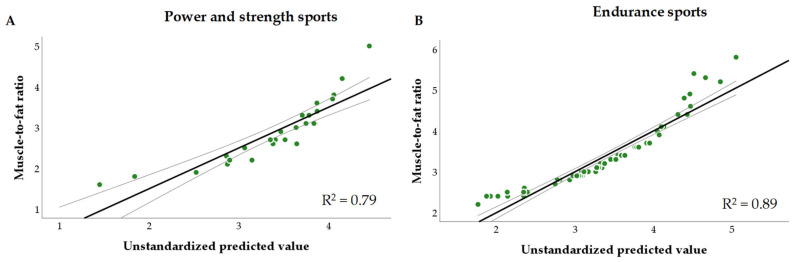
The association between MFR and body composition components, namely, SMM (in kg) and BF (in kg). (**A**) Multiple linear regression model A: F (2.23) = 42.4, *p* < 0.0001, R^2^ = 0.79. (**B**) Multiple linear regression model B: F (2.62) = 232.3, *p* < 0.0001, R^2^ = 0.89.

A more detailed data analysis revealed the relationship between MFR and body composition components such as SMM (in kg) and BF (in kg). Figure 2 shows the two multiple linear regression models, namely, A and B, included in the analysis.

The linear regression analysis showed that female athletes participating in strength and power exercises had higher MFRs in an inverse association with excess adiposity (β −0.1, 95% CI: −0.15; −0.07). In the meantime, the female endurance athletes with higher MFRs depended on both lower fat mass (β −0.27, 95% CI: −0.3; −0.2) and higher skeletal muscle mass (β 0.08, 95% CI: 0.06; 0.1). Thus, in both cases, irrespective of the type of sports, an excessive body fat percentage was potentially factored as a negative outcome for maintaining the optimal body composition, and was considered a meaningful variable likely to influence physical fitness in female athletes. In addition, if the study subgroups by sports branches were heterogeneous according to the characteristics of body composition, the logistic regression model should be adjusted for sports branches, considered the main confounding variable during the forthcoming steps of the data analysis.

### 3.2. Energy, Macronutrient, and Micronutrient Intakes

Table 2 reveals the reported usual mean intakes of energy, carbohydrates, fats, proteins, and B vitamins in professional female athletes. 

For females, the reported average intakes of energy, carbohydrates, protein, and fat were 2475 ± 864 kcal/day, 5.1 ± 2.4 g/kg/day, 1.5 ± 0.5 g/kg/day, and 36.7 ± 7.5%, respectively. Furthermore, the mean amounts of B vitamins consumed by female athletes were reported as follows: vitamin B_1_ (1.6 ± 0.8 mg/day), vitamin B_2_ (2.2 ± 1.1 mg/day), vitamin B_3_ (21 ± 8.5 mg NE/day), vitamin B_6_ (2.6 ± 0.9mg/day), vitamin B_9_ (216.4 ± 86.4 mcg/day), vitamin B_12_ (4.5 ± 4.0 mcg/day).

**Figure 3:**
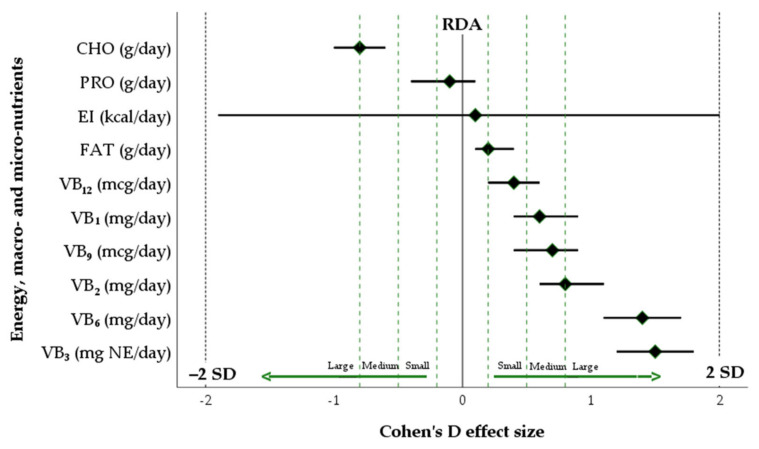
The measure of Cohen’s D reporting the effect size of the mean differences in dietary macro- and micronutrient intakes after comparing them to the RDAs. CHO—carbohydrates; FAT—fat; EI—energy intake; V—vitamin; NE—niacin equivalent; RDA—recommended dietary allowance; SD—standard deviation.

As indicated in Figure 3, when comparing nutrient intakes to the RDAs, only a carbohydrate-deficient diet was identified in female athletes. Despite a slightly positive energy balance (∆ 95 kcal/day, 95% CI: −1607; 217), as compensation led by the insufficient intake of carbohydrates, a high-fat diet consisting of more than 36% of the total calories was consumed by the study participants. 

### 3.3. Association between Nutrient Intakes and Body Fat Mass

**Figure 4:**
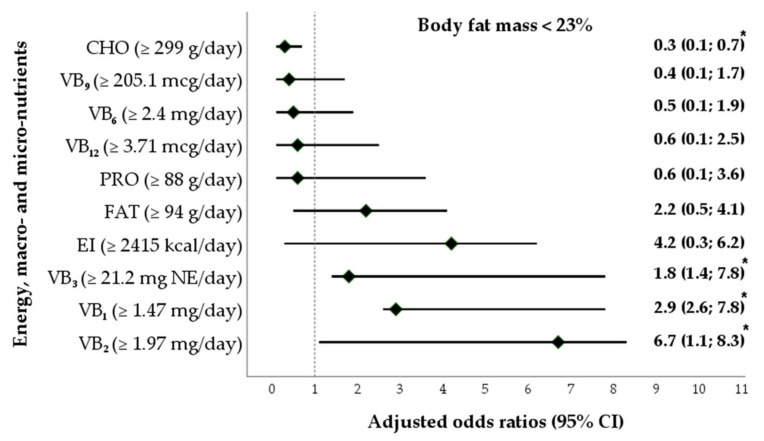
The association between the intake of energy, macronutrients, and B vitamins (B_1_, B_2_, B_3_, B_6_, B_9_, B_12_) and body fat mass as dependent variables in female athletes (multivariate analyses). CHO—carbohydrates; FAT—fat; EI—energy intake; V—vitamin; NE—niacin equivalent; V—vitamin; aOR—adjusted odds ratio = e^β^; 95% CI—95% confidence interval; *—*p*-value < 0.05.

As shown in Figure 4, the analysis included one multivariate logistic regression model. After adjustment for the sports disciplines, lower intakes of carbohydrates were associated with lower odds of having a lower body fat percentage (aOR 0.3, 95% CI 0.1; 0.7). In addition, the assessment of the sample of female athletes showed that the aORs for the lower percentage of body fat were related to higher intakes of vitamin B_1_ (aOR = 2.9, 95% CI 2.6; 7.8), vitamin B_2_ (aOR 6.7, 95% CI 1.1; 8.3), and vitamin B_3_ (aOR 1.8, 95% CI 1.4; 7.8).

Figure 4 reflects that the adjusted odds ratios (aORs) and their 95% confidence intervals (CIs) (min; max) for the macro- and the specified micronutrient intake predicted lower body fat percentage. If aORs, including values of 95% CI, were higher than 1 or lower than 1, a positive or negative association between the variables was determined, respectively. If the aORs crossed the dotted line, there was no association between the variables under analysis. The magnitude of the aORs was not interpreted in an ascending or a descending manner.

The reference category was body fat mass ≥ 23%. The logistic regression model was adjusted for the sports branches of the female athletes. The measure of goodness of fit in the logistic regression analysis was R^2^_N_ = 0.28; *p* = 0.28.

## 4. Discussion

While conducting this cross-sectional study, we examined the association between the variation in body fat mass magnitude and the intake of nutrients, including carbohydrates, fat, and B vitamins, in a cohort of highly trained female athletes from Lithuania.

Our findings showed that despite a slightly positive energy balance (∆ 95 kcal/day), carbohydrate-deficient diets along with higher intakes of vitamin B_1_, vitamin B_2,_ and vitamin B_3_ from food were associated with lower body fat percentage in female athletes. The results of similar studies carried out in other countries, such as Canada [23,24], Poland [25,26], Brazil [27], Portugal [28], Australia [29], Spain [30], the Netherlands [21], the United Kingdom [32], Mexico [33], the United States [34], and Indonesia [35], have shown that athletes regularly consumed high-fat foods (ranging from 27% to 37%), which easily contributed to the insufficient intake of carbohydrates (ranging from 2.4 g/kg to 6.6 g/kg). The results of our study were also consistent with those obtained by other researchers and revealed an inadequate intake of carbohydrates (~5 g/kg vs. 7–10 g/kg), notwithstanding the fact that Lithuanian elite female athletes were engaged in physical activities of 2–3 h training with moderate–high intensity. 

It should also be highlighted that there is some scientific evidence referring to the potential benefits of low-carbohydrate diets in cases where liver and muscle glycogen stores in athletes have not been fully replenished as a result of exercise [68,69,70]. Therefore, such conditions may be potentially associated with long-term bodily adaptations to augment fatty acid oxidation rates via the promotion of mitochondrial biogenesis [71]. In our case, the participation of female athletes in physical activity accompanied by the training method ‘training low’ was spontaneous. It is worth mentioning that ‘training low’ as a training–nutrition strategy [72], designed to purposefully deplete muscle glycogen stores due to the reduced availability of exogenous carbohydrates along with a peptide hormone, namely, insulin, may contribute to the occurrence of alterations in lipid metabolism and trigger both lipolysis and the mobilization of fatty acids. Although the relationship between the usual isocaloric low-carbohydrate diets and lower body fat percentage was confirmed by our study, major concerns remain due to the inadequate intake of dietary carbohydrates around exercise, in line with the possibility of contributing to rapid fatigue in the central nervous system and a negative impact on the immune system [73] and aerobic fitness [74,75]. For these reasons, low-carbohydrate diets have still not been recommended for professional athletes.

Low-carbohydrate diets were associated with an increased fat intake according to our study sample. Although high-fat diets do not adversely affect traditional lipid parameters such as plasma triglycerides and low- and high-density lipoprotein cholesterol profiles in athlete populations [76], there is scientific evidence revealing a positive correlation between the fat intake and the increased levels of circulating homocysteine acting as a cardiometabolic risk factor [77,78]. Thus, our study speculates on a meaningful issue related to the nutritional goal to be assigned to the athletic population as a recommendation for a change from a high-fat isocaloric diet consisting of ~36% of total calories from fats to a high-carbohydrate diet. Furthermore, in line with this recommendation, it must be highlighted that following scientific research, the decrease in circulating homocysteine was also related to the increased consumption of B vitamins, such as B_2_, B_6_, B_9,_ and B_12_, which were recognized as cofactors of enzymes engaged in homocysteine metabolism [79,80,81,82]. 

Finally, B vitamins play a significant part in biological processes [83] and energy metabolism [39,84]. If the consumed amount falls below the RDA for B vitamins (B_1_, B_2_, B_6_, and B_9_), the physical body may suffer from a micronutrient deficiency likely to lead to the disturbances in energy metabolism, and consequently, cause fat accumulation as well as potentially increased adipose tissue [44,45,46,85,86,87]. In our case, the levels of B vitamins consumed by female athletes did not differ from the RDAs; therefore, these findings were consistent with current studies reporting a good status for vitamins B_6_, B_9_, and B_12_ in highly active females [88,89]. Also, the multivariate logistic regression analysis disclosed an association between a higher intake of B vitamins, namely, B_1_, B_2,_ and B_3_, and lower body fat among female athletes. The explanatory mechanisms between the development of body fat and higher intakes of vitamin B_1_, vitamin B_2_, and vitamin B_3_ have not yet been clarified in the athletic population. The predictable and only possible pathways have been speculated to explore the relationship between vitamin B levels and the progress of body fat mass in the non-athletic population [39,84,90,91,92]. 

## 5. Strengths and Limitations

Our study has some strengths, as it prioritized the nutritional care of female athletes playing different sports. This study has, for the first time, contributed to the evidence of a relationship between variation in body fat mass magnitude and the intake of selected nutrients from food, specifically, carbohydrates and vitamins B_1_, B_2_, and B_3_. Consequently, athletes pursuing long-term body composition goals (in terms of the optimal muscle-to-fat ratio) can benefit from food sources of vitamin B_1_ and carbohydrates, including green vegetables, whole-grain products, and rice bran rather than meat [93]. On the other hand, the intake of vitamins B_2_ and B_3_ in conjunction with protein may be increased by eating a diet rich in foods such as milk, eggs, and lean meats [93].

This study has some limitations. First, it would be more accurate to detect vitamin B levels in blood serum or whole blood; therefore, further studies should lay emphasis on the association between plasma concentrations of B vitamins and the variation in body fat levels in the athletic population. Second, as genetics and sport selection are confounding variables when it comes to establishing what determines body fat, it is not possible to conclude that (a) a lower-carbohydrate diet will lead to lower body fat, and (b) higher intakes of vitamin B_1_, vitamin B_2_, and vitamin B_3_ lead to increased body fat. On the condition that the study was cross-sectional in design and unfitted to figure out cause-and-effect, there remains a necessity for further randomized experimental studies to validate the effect of B vitamin intake on adipose tissue development and metabolism. Third, as our study represented only elite athletes, the estimated representative sample size was relatively small. Finally, the main study participants were female athletes; therefore, sports professionals should be cautious when extrapolating the empirical data to male athletes or non-athletic populations.

Taking into account that current research on vitamin B and fat metabolism is restricted and incompatible, further studies are needed to confirm the relationship between the intake of different B vitamins and the adiposity levels in a wider athletic population. Additionally, this study supports further hypotheses in future research that rely on how the supplementation with B vitamins can possibly result in a reduction in body weight gain, thus promoting potential benefits to professional athletes following long-term goals for body composition and fitness [46,75].

## 6. Conclusions

As an important part of triggering the adaptations to endurance or power and strength training, elite female athletes did not maintain a diet high in carbohydrates. The Western-like low-carbohydrate (~5 g/kg/day) isocaloric diet, consisting of at least 36% of total calories from fats, was consumed by the sample of female athletes. 

Despite a slightly positive energy balance (∆ 95 kcal/day), the carbohydrate-deficient diet, along with higher intakes of vitamin B_1_, vitamin B_2_, and vitamin B_3_ from food, was associated with a lower percentage of body fat in our cohort of female athletes. Therefore, when female athletes with a higher body fat percentage attempt to achieve optimal body composition by limiting carbohydrates, they should be cognizant of potentially shortening their intake of protein and specific B vitamins.

## Figures and Tables

**Table 1:** Body composition of female athletes according to sport branches.

Height and Body Components	Power and Strength Sports (n = 26)	Endurance Sports (n = 63)
H, m	1.7 ± 0.1	1.7 ± 0.2
BM, kg	67.4 ± 14.3 *	59.7 ± 7.5
FFM, kg	50.1 ± 7.9 *	46.3 ± 4.7
FFM%	75.2 ± 4.9 *	77.6 ± 3.7
SMM, kg	46.2 ± 7.2 *	42.8 ± 4.3
SMM, %	69.4 ± 4.8 *	72.1 ± 3.6
BF, kg	17.6 ± 7.1 **	13.4 ± 3.5
BF, %	24.9 ± 4.8 *	22.1 ± 3.6
MFR	2.9 ± 0.8	3.4 ± 0.8 *

Data are presented as means ± SDs; SD—standard deviation; BM—body mass; FFM—fat-free mass; SMM—skeletal muscle mass; FM—fat-free mass; BMI—body mass index; MFR—muscle-to-fat ratio; H—height; *—*p*-value < 0.05; **—*p*-value < 0.01.

**Table 2:** Nutrient intakes in female athletes according to sport branches.

Energy and Macro- and Micronutrients	Power and Strength Sports (n = 26)	Endurance Sports (n = 63)	RDAs	∆ (95% CI)
EI, kcal/day	2457 ± 628	2480 ± 948	2501 ± 188	95 (−1607; 217)
CHO, g/kg/day	4.7 ± 1.7	5.2 ± 2.6	7–10	−1.9 (−2.4; −1.5)
PRO, g/kg/day	1.4 ± 0.4	1.5 ± 0.6	1.2–2.0	−0.1 (−0.2; 0.0)
FAT%	36.1 ± 7.4	36.8 ± 7.8	25–35	1.6 (0.1; 3.2)
VB_1_, mg/day	1.3 ± 0.5	1.7 ± 0.9	1.1	0.5 (0.3; 0.6)
VB_2_, mg/day	2.2 ± 0.9	2.2 ± 1.2	1.3	0.9 (0.7; 1.2)
VB_3_, mg NE/day	20 ± 4.6	21.5 ± 9.6	8	13 (11.2; 14.8)
VB_6_, mg/day	2.6 ± 0.5	2.6 ± 1.1	1.3	1.3 (1.1; 1.5)
VB_9_, mcg/day	219.7± 71.5	215.1 ± 92.4	200	16.4 (−1.8; 34.6)
VB_12_, mcg/day	5.9 ± 3.3	3.9 ± 2.3	3	1.5 (0.7; 2.3)

Data are presented as means ± SDs; SD—standard deviation; EI—energy intake; CHO—carbohydrates; PRO—protein; FAT—fat; V—vitamin; NE—niacin equivalent; RDA—recommended dietary allowance: average daily level of intake sufficient to meet the nutrient requirements for nearly all (97–98%) healthy individuals; ∆—the mean differences in dietary macro- and micronutrient intakes compared to the RDAs; 95% CI—95% confidence interval.

## Data Availability

Data is contained within the article.

## Nomenclature

Acronyms and terminology

**Term**	**Definition**
∆	Delta
aOR	Adjusted Odds Ratio
BIA	Bioelectrical Impedance Analysis
BM	Body Mass
BF	Body Fat
CHO	Carbohydrates
CI	Confidence Interval
D	Cohen’s D Effect Size
DRI	Dietary Reference Intake
EAR	Estimated Average Requirement
EER	Estimated Energy Requirement
EI	Energy Intake
EN-ISO	International Organization for Standardization adopted by the European Union
FAT	Fat
H	Height
FFM	Fat-Free Mass
R^2^_N_	Nagelkerke R^2^
RDA	Recommended Dietary Allowance
RQ	Research Question
kHz	Kilohertz
MET	Metabolic Equivalent
MFR	Muscle-to-Fat Ratio
NE	Niacin Equivalent
*p*	*p*-value
PRO	Protein
RMR	Resting Metabolic Rate
SD	Standard Deviation
SMM	Skeletal Muscle Mass
SPSS	Statistical Package for the Social Sciences
TEE	Training Energy Expenditure
USA	The United States of America
V	Vitamin
vs.	Versus
